# Currarino syndrome as an incidental radiologic finding in a patient with acute flank pain: A case report^☆^

**DOI:** 10.1016/j.radcr.2022.05.039

**Published:** 2022-06-17

**Authors:** Mehdi Ghaderian Jahromi, Sara Haseli, Pooya Iranpour, Amir Mohammad Nourizadeh

**Affiliations:** a Medical Imaging Research Center, Department of Radiology, Shiraz University of Medical Sciences, Shiraz, Iran; b Chronic Respiratory Diseases Research Center, National Research Institute of Tuberculosis and Lung Diseases (NRITLD), Shahid Beheshti University of Medical Sciences, Tehran, Iran

**Keywords:** Currarino syndrome, Acute flank pain, Presacral mass, Horseshoe kidney, Radiology, Neurosurgery

## Abstract

Currarino syndrome is an autosomal dominant hereditary disease defined as a triad of anorectal abnormality, sacral dysgenesis, and a presacral mass, primarily an anterior sacral meningocele. It is often seen in children and considered rare in adults. It is mostly found as an incidental finding. We present a 21-year-old man who presented with acute flank pain. He had a history of Hirschsprung's disease and therefore had undergone surgery in his infancy. He also had a history of prolonged constipation and had an episode of admission due to suspected obstruction. On physical examination, he had a severe costovertebral angle tenderness. urine exam revealed microscopic hematuria. Laboratory tests were otherwise unremarkable. Computed tomography scan revealed renal stones as well as a horseshoe kidney. Incidental findings included a large simple cystic structure in the presacral area suggestive of an anterior meningocele and sacral dysgenesis associated with scimitar sacral appearance. These findings suggested a diagnosis of Currarino syndrome. Urinary complications of this disease are reported in few articles. An important takeaway note for physicians is to have a high level of suspicion when encountering patients with gastrointestinal, neurologic, or urologic signs and symptoms and consider a thorough history taking and physical examination alongside proper imaging evaluation.

## Introduction

Currarino syndrome (CS) is an autosomal dominant hereditary disease defined as a triad of anorectal abnormality, sacral dysgenesis, and a presacral mass, primarily an anterior sacral meningocele. To the best of our knowledge, less than 400 cases of this syndrome have been reported in the medical literature to this date, and most cases are diagnosed before adulthood. This syndrome is considered rare in adult patients [1,2]. In some adult cases, it may remain asymptomatic and be found as an incidental finding. In contrast, in other cases, it may cause constipation, rectal fullness, and abdominal pain due to extensive enlargement of the sacral mass [3]. This could increase the possibility of missing the patient if asymptomatic or misdiagnosing the patient for gastrointestinal problems if the patient presents with aforementioned signs and symptoms.

We report a case of Currarino Triad in an adult male, which was diagnosed as an incidental radiologic finding.

## Case report

**Fig. 1:**
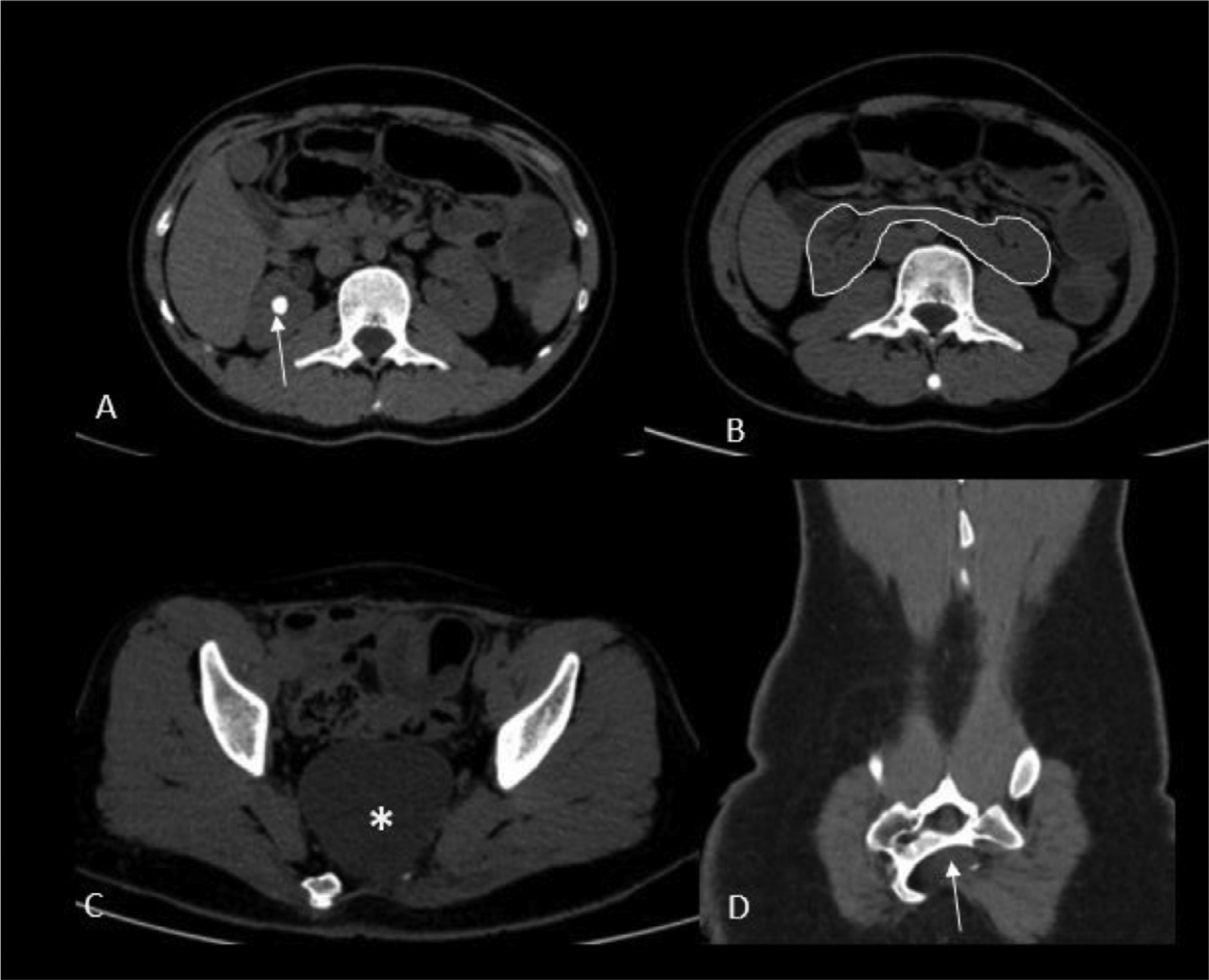
Axial non-enhanced CT scan shows right sided radiopaque renal stone (arrow, A). Horseshoe kidney (B) anterior sacral meningocele (*, C), and sacral dysgenesis (arrow, D).

A 21-year-old man presented to the clinic with acute right flank pain, nausea and vomiting, and no constipation, or abdominal distention. He had a history of Hirschsprung's disease, and therefore, had undergone surgery in his infancy. He also had a history of prolonged constipation and had an episode of admission due to suspected obstruction but had been discharged without any complications. On examination, he had a blood pressure of 125/80, a heart rate of 93, a respiratory rate of 20, and a temperature of 37.8. On physical examination, he had severe costovertebral angle tenderness. Neurologic examination was normal. His history and physical examination were otherwise unremarkable. Urine exam revealed microscopic hematuria. Laboratory tests were otherwise unremarkable. With a probable diagnosis of renal colic, the patient underwent an abdominal computed tomography (CT) scan. The scan showed horseshoe kidneys and renal stones in the right kidney, confirming the diagnosis. Several incidental findings also included a large simple cystic structure in the presacral area suggestive of an anterior meningocele and sacral dysgenesis associated with scimitar sacral appearance (as shown in Fig. 1). These 2 latter radiologic findings, along with a previous history of anorectal abnormality (Hirschsprung's disease in this case), suggested a diagnosis of CS for this patient. The patient was referred to the urology and neurosurgery clinic for further evaluation of the renal stone and presacral mass.

## Discussion

CS was first described by Currarino et al [4], in 1981, as a triad of congenital anorectal stenosis, a defect in sacral bone, and a presacral mass. To the best of our knowledge, less than 400 cases of this rare syndrome have been reported since then [1,5]. CS is a hereditary autosomal dominant disease with mutations in the homeobox gene HLXB9 located on chromosome 7q36, encoding the nuclear protein HB9. Mutations of this gene are found in approximately 90% of familial CS cases and 30% of sporadic CS cases. The female-to-male ratio in sporadic cases is 3:1, whereas in familial cases, it is 1:1. There is no reported genotype-phenotype correlation [1,^5^,6]. CS is not a common diagnosis in adult patients since most symptomatic cases are diagnosed in early childhood. Adult patients may be asymptomatic or have gastrointestinal, urinary, or neurologic symptoms [3,^7^,8]. Our patient did not have any gastrointestinal or neurologic symptoms. He underwent a CT scan due to acute flank pain associated with renal stone, and the Currarino triad was incidentally found.

Patients can be asymptomatic or present with various signs and symptoms, including constipation, abdominal distention, abdominal pain, nausea and vomiting, rectal fullness, abdominal and pelvic masses, low back pain, headaches, fever, arthralgia, recurrent meningitis, and urinary tract infection [1,^3^,^5^,[8], [9], [10], [11], [12], [13], [14], [15]. The most common symptom is chronic constipation. The cause of constipation in CS is still unknown [9]. Although CS comprises the triad of anorectal abnormalities, sacral defect, and a presacral mass, the phenotypic presentation of many patients, lacks all 3 features [9]. There are also other malformations in different organs associated with CS. Lynch et al [16] reported that urinary malformations in CS include horseshoe or duplex kidney, duplex ureter, vesicoureteral reflux, secondary hydronephrosis, neurogenic bladder, recurrent urinary tract infections, and urinary incontinence. There have been several reports of patients with CS who also had urologic complications [17], [18], [19]. Duru et al [9] reported a patient presenting with low back pain, weakness in lower extremities, and urinary incontinence diagnosed with CS via MRI. Shin et al reported a patient who presented with a pelvic mass and chronic constipation. Further imaging investigations revealed a right kidney that was small in size and had calyceal blunting and hypertrophy of the left kidney [1]. Versteegh et al [14] reported a patient who presented with periodical headaches and urinary incontinence. Further imaging investigations revealed a double bladder. To the best of our knowledge, horseshoe kidney has been the least reported complication among the urologic complications. Our patient's radiologic findings include a complete Currarino triad as well as a horseshoe kidney, which favors the association between CS and urologic complications such as horseshoe kidney.

Imaging techniques such as ultrasonography, plain radiography, CT scan, and MRI are useful for diagnosing this condition. Pelvic radiography efficiently diagnoses the sacral deformities, and the physician should consider the possibility of CS. Further imaging investigations, such as CT scan or MRI, should be performed to reveal any presacral mass, confirming the diagnosis of CS [5]. MRI is often the modality of choice specially when looking for the presence of a presacral mass, associated spinal cord anomalies, and evaluating abdominopelvic organs, distinguishing the presacral mass and other abdominal structures, and other spinal cord related injuries [1,8]. Our patient underwent a CT scan, revealing scimitar sacral deformity and a presacral meningocele. Together with a history of Hirschsprung's disease, Currarino triad was present in this patient, and he was diagnosed with CS.

Management of CS mainly depends on the presence of a presacral mass and/or anorectal malformations [5]. Initial management should focus on treating anorectal malformations, which usually includes surgical management, although some studies suggest conservative management for milder cases [9]. On the other hand, surgical management is usually the preferred treatment for presacral masses as they cause complications, including neurologic and gastrointestinal. Some studies have proceeded with a more conservative approach in patients with meningocele and have achieved acceptable results with fewer postoperative complications [18]. Our patient had undergone reconstructive surgery in his infancy due to Hirschsprung's disease. Unfortunately, we still have no information on the possible neurosurgical management of the patient since he is still in the evaluation process.

## Conclusion

Currarino syndrome is a hereditary disease defined as the triad of anorectal abnormality, sacral malformations, and a presacral mass. While the most common clinical presentation is constipation, adult cases may be asymptomatic and CS could be an incidental finding. This highlights the importance of thorough history taking and imaging evaluation, preferably MRI, as the primary tools for diagnosing this syndrome. Management of this condition depends on the severity of the disease but mainly includes surgical management of anorectal malformations and presacral masses.

## Statement of ethics

This research was approved by the “Faculty Research Ethics Committee of Shahid beheshti University of Medical Sciences” and “Faculty Research Ethics Committee of Iran National Committee for Ethics in Biomedical Research” with the code of IR.SBMU.NRITLD.REC.1400.083.

## Author contributions

Both AM.N. and M.G. wrote the initial draft of the manuscript. AM.N, M.G., S.H.,and P.I. reviewed and participated in the final version of manuscript.

## Patient consent statement

The authors have obtained a written informed consent from the patient to publish his case (including publication of images).

## Data availability statement

All data generated or analyzed during this study are included in this article. Further enquiries can be directed to the corresponding author.
